# A Combination of *Lacticaseibacillus paracasei* CECT 30660 and *Bifidobacterium longum* subsp. *infantis* CECT 7210 Cell-Free Supernatants Reduces LPS-Induced Preterm Birth and Systemic Inflammation in Pregnant Mice

**DOI:** 10.3390/nu17213429

**Published:** 2025-10-31

**Authors:** Sergio Quesada-Vázquez, Maria Cristina De Almagro García, Gloria Cifuentes-Orjuela, Anna Antolín, Juan María Alcaide-Hidalgo, Jesús Jiménez, Francesc Puiggròs, Antoni Caimari, Fàtima Sabench, Josep M. Del Bas, Xavier Escoté, José Antonio Moreno-Muñoz

**Affiliations:** 1 Unitat de Nutrició i Salut, Centre Tecnològic de Catalunya, Eurecat, 43204 Reus, Spain; sergio.quesada@ordesalab.com (S.Q.-V.); anna.antolin@eurecat.org (A.A.); juanmaria.alcaide@eurecat.org (J.M.A.-H.); francesc.puiggros@eurecat.org (F.P.); 2 Laboratorios Ordesa S.L, Parc Científic de Barcelona, Edifici Hèlix, Carrer Baldiri Reixac 15-21, 08028 Barcelona, Spaingloria.cifuentes@ordesalab.com (G.C.-O.); jesus.jimenez@ordesalab.com (J.J.); 3 Biotechnology Area, Centre Tecnològic de Catalunya, Eurecat, 43204 Reus, Spain; antoni.caimari@eurecat.org (A.C.); josepm.delbas@urv.cat (J.M.D.B.); 4 Grup de Recerca en Cirurgia del IISPV, Universitat Rovira i Virgili, 43204 Reus, Spain; fatima.sabench@urv.cat; 5 Nutrigenomics Research Group, Department of Biochemistry and Biotechnology, Institut d’Investigació Sanitària Pere Virgili (IISPV), Center of Environmental, Food and Toxicological Technology (TecnATox), Universitat Rovira i Virgili, 43007 Tarragona, Spain; 6 Nutrition and Metabolic Health Research Group, Department of Biochemistry and Biotechnology, Institut d’Investigació Sanitària Pere Virgili (IISPV), Center of Environmental, Food and Toxicological Technology (TecnATox), Universitat Rovira i Virgili, 43204 Reus, Spain; 7 Centro de Investigación Biomédica en Red de Fisiopatología de la Obesidad y Nutrición, Instituto de Salud Carlos III, 28029 Madrid, Spain

**Keywords:** *Bifidobacterium longum*, *Lacticaseibacillus paracasei*, cell-free supernatant, preterm birth, inflammation, cytokines, LPS, murine model, pregnancy, microbial metabolites

## Abstract

**Background/Objectives.** Preterm birth (PTB), affecting approximately 11.1% of pregnancies globally, often results from inflammation at the maternal–fetal interface triggered by microbial or immune dysregulation. This study investigates the efficacy of cell-free supernatant derived from *Bifidobacterium longum* subsp. *infantis* CECT 7210 and *Lacticaseibacillus paracasei* CECT 30660 in mitigating inflammation-induced PTB in a murine model. **Methods.** Lipopolysaccharide (LPS) was administered to induce preterm labor and systemic inflammation, mimicking infection-related PTB. **Results.** The results demonstrated that combined administration of CECT 7210 and CECT 30660 cell-free supernatants reduced preterm deliveries from 85.6% to 42.8% in mice and significantly attenuated systemic and intrauterine proinflammatory cytokines, including TNF-α and IL-6, in maternal plasma and myometrial tissues. Importantly, this anti-inflammatory effect was independent of maternal progesterone or oxytocin levels, suggesting a direct modulation of immune responses in this animal model. The cell-free supernatant combination also inhibited the growth of pathogenic bacteria, including *Streptococcus agalactiae*, highlighting its antimicrobial potential. **Conclusions.** This study underscores the potential of CECT 7210 and CECT 30660 cell-free supernatants as a therapeutic strategy to reduce the risk of PTB by targeting inflammation pathways. The findings pave the way for further preclinical and clinical research to validate the efficacy of these cell-free supernatants in preventing PTB and associated complications, offering a promising alternative to traditional probiotic approaches.

## 1. Introduction

Microbial communities play a critical role in promoting homeostasis in the vagina and preventing the colonization of pathogenic bacteria. Lactobacilli are considered the dominant inhabitants of the human vagina and are responsible for its health [1]. The prevailing hypothesis holds that vaginal *Lactobacillus* species promote a protective environment by lowering the pH below 4.5 through the production of lactic acid and by competing for nutrients and space with other bacteria. A symbiotic relationship is established between these bacteria and the host. *Lactobacillus* also produces other metabolites, such as bacteriocins and hydrogen peroxide, which may contribute to the inhibition of the growth of other microorganisms [2,3]. The Nugent score [4] allows the evaluation of a woman’s vaginal health based on the number of lactobacilli present in the woman’s vagina; the greater the number of lactobacilli, the better the woman’s vaginal health, while low or no numbers, along with increases in *Gardnerella*, *Bacteroides* spp., and Gram-negative bacteria, are indicative of poor health.

Lactobacilli can also inhibit pathogen colonization by competing for host cell receptors used by urogenital pathogens such as *Gardnerella vaginalis*, *Neisseria gonorrhoeae*, *Candida albicans*, *Staphylococcus aureus*, group B *Streptococcus* species (GBS) such as *Streptococcus agalactiae*, *Pseudomonas aeruginosa*, *Escherichia coli*, and *Prevotella bivia* [5,6]. Furthermore, some lactobacilli co-aggregate with pathogens, thereby inhibiting attachment to host cells and allowing more efficient clearance [7,8,9]. The vaginal microbiota can also undergo changes during pregnancy since alpha diversity decreases as gestation progresses, where *L. crispatus* and *L. iners* dominate the vaginal microbiota [10]. However, the presence of *L. gasseri* or *L. iners* in pregnant women is related to a higher risk of suffering from vaginitis since the proportion of these lactobacilli decreases throughout the gestation trimesters, while in women colonized by *L. crispatus* this ratio is maintained [11].

These lactobacilli species are closely related and are thought to perform similar ecological roles in the vaginal environment, such as lactic acid production [12]. Mammalian cells, including vaginal epithelial cells, produce only the L-isomer of lactic acid. Instead, lactic acid-producing bacteria produce both isomers of D- and L-lactic acid [13]. L-lactic acid, in addition to its role in influencing vaginal acidity, has specific immune properties, such as stimulation of the interleukin 23 (IL-23)/IL-17 T cell pathway and activation of lymphocytes [14,15,16]. *L. crispatus* is the one that produces the greatest amount of total lactic acid, followed by *L. gasseri*. On the other hand, *L. jensenii* exclusively produces D-lactate and *L. iners* only produces the other isomer [17].

Preterm birth (PTB) is described as birth before the 37th week of gestation, and the worldwide prevalence of this condition is about 11.1% of all pregnancies, involving up to 15 million pregnancies each year globally [18,19]. Being born prematurely can lead to adverse long-term health outcomes, such as neonatal death, a high risk of early-life infections, and preterm-induced disorders later in life [18]. Hence, PTB is a risk factor that has an impact on development in adult life [19]. Several factors can cause PTB, and it goes from endogenous factors (short cervical length, maternal nutrition depletion, etc.) to exogenous factors (pollution, drug consumption, etc.) [19].

An important contributing factor to both infection-mediated PTB and spontaneous PTB is inflammation. Inflammation may be caused by infection through microorganisms’ invasion of the uterus via ascension through the vagina or via the placenta [20,21], or it may often originate from the gut [22]. The impact of nutrition and gut microbiota balancing in early life is well described, which may condition the risk of suffering from inflammatory diseases [23]. It is described that almost half of all PTB are provoked or triggered by inflammation at the fetal–maternal interface, ending in preterm labor or rupture of membranes, contributing to the development of diverse preterm-induced diseases [22]. Cytokines play a key role in PTB, acting as regulators of the innate and adaptive immune systems. There is existing evidence on the association of levels of several cytokines with PTB [24,25]. Some studies suggested elevated intrauterine and circulating levels of proinflammatory cytokines are strongly associated with PTB [26,27]. Interferon-γ (IFN-γ) and tumor necrosis factor alpha (TNF-α), which are produced by fetal T cells, cause contractility of myometrial cells, a key factor in parturition [25]. Increased levels of interleukins (IL)-1α, IL-1β, and IL-6 have been associated with fetal inflammatory response to maternal infection, increasing risk status for PTB [28,29]. However, IL-10 showed an anti-inflammatory effect that diminished the inflammatory response, lowering PTB risk [30]. It has also been seen that elevated levels of progesterone, a sexual hormone, play a role in PTB prevention and exert significant anti-inflammatory effects in pregnancy, maintaining uterine quiescence [31,32]. Moreover, oxytocin is a hormone whose response is linked to progesterone. Oxytocin is a potent stimulant of uterine contractions and could be an important factor in PTB, including novel roles for oxytocin as an inflammatory mediator [33,34]. To study the effect of inflammation in PTB, some animal models were developed, and the most commonly used approach is to cause inflammation through lipopolysaccharide (LPS) injection into the uterus [35]. LPS, a component of the membrane of Gram-negative bacteria [36], binds toll-like receptor 4 (TLR4) and activates the nuclear factor κ light-chain-enhancer of activated B cells (NF-kB) pathway, triggering proinflammatory cytokine and chemokine release. In this study, we administered LPS to pregnant CD-1 mice with the objective of developing an inflammation-associated PTB [35].

There is a hypothesis that PTB might be triggered by maternal and placental dysbiosis. With the aim of treating inflammatory conditions like bacterial vaginosis or dysbiosis in the intestinal tract or placenta, probiotics, which are live microorganisms, have been demonstrated to have a health benefit on the host [37]. Some probiotics from the *Lactobacillus* and *Bifidobacterium* genus showed capacity to modulate immune response and anti-inflammatory properties in the neonatal period [20,38]. Genus *Bifidobacterium* colonizes the newborn gut within the first days after birth and has a vertical transmission from the mother to the baby (vagina, GI tract, or breast milk). Actually, the subspecies *Bifidobacterium infantis* dominates the gut microbiota of breastfed infants [39]. Antibiotic administration as a strategy to prevent PTB has shown ineffective results, promoting resistant bacterial strains and a decrease in *Lactobacillus* and *Bifidobacterium* abundance [38]. An innovative alternative to probiotics is postbiotics, which are functional bioactive compounds from fermentation, including microbial cells, cell constituents, and metabolites that might promote health [40]. The ISAPP consensus defines postbiotics as preparations containing inanimate microorganisms and/or their components [41], with or without associated metabolites, that confer health benefits to the host. In addition, a probiotic strain isolated from particular population groups (breast-fed infants), *Bifidobacterium longum* subsp. *infantis* CECT 7210, commercially called *B. infantis* IM1^®^ (Laboratorios Ordesa S.L, Parc Científic de Barcelona, Edifici Hèlix, Carrer Baldiri Reixac 15-21, 08028 Barcelona), has been shown in several studies to provide some beneficial effects in different health complications through microbiota enrichment and protection against pathogens [42,43,44], demonstrating anti-inflammatory effects [42]. Moreover, CECT 7210 has shown potential features to be a promising probiotic in infant foods [23].

The effect of CECT 7210 and a novel strain of *Lacticaseibacillus paracasei* CECT 30660 (Laboratorios Ordesa S.L.) on PTB and inflammation in a pregnant CD-1 mouse model is unknown. In this study, we tested the hypothesis that these cell-free supernatants generated from bacterial strains CECT 7210 and CECT 30660 (alone or in combination) will attenuate LPS-induced PTB and reduce systemic and intrauterine immune markers in LPS-treated mice. In addition, we evaluated whether the effect of cell-free supernatant on LPS-induced PTB is dependent on changes in maternal plasma progesterone or maternal and amniotic oxytocin.

## 2. Materials and Methods

### 2.1. Isolation and Preliminary Characterization of Bacterial Candidates

Lactobacilli strains were originally isolated from vaginal exudate samples collected with sterile swabs of healthy women. Human ethical clearance for sample col-lection was obtained as previously described [43]. Briefly, bacterial colonies were isolated under sterile conditions by seeding them on plates of different culture media (MRS, Tomato, and Rogosa Agar). The colonies were selected for the different morphologies observed, taking into account shape, surface, and edge. The selected colonies were assigned a colony alphanumeric code and incubated at 37 °C for 24 h. In the event that there was not enough growth, they were allowed to incubate longer, a maximum of 72 h. With the grown colonies, Gram staining was performed, where the bacteria and the cellular morphology of the bacteria were determined, selecting colonies that presented morphology of elongated bacilli, with Gram-positive bacteria.

### 2.2. Bacterial Strains and Growth Conditions

*B. longum* subsp. *infantis* CECT 7210 was grown using MRS-C medium (de Man Rogosa Sharpe broth [Merck KGaA, Darmstadt, Germany] supplemented with 0.05% [*w*/*v*] cysteine [Sigma-Aldrich, St. Louis, MO, USA]) and incubated anaerobically by means of an Anaerocult A system (Merck KGaA, Darmstadt, Germany) at 37 °C for 36 to 72 h. The *Lactobacillus* strains were grown on MRS (Merck KGaA, Darmstadt, Germany) medium and incubated anaerobically at 37 °C for 17 to 24 h. *Streptococcus agalactiae* was grown using nutrient broth (Merck KGaA, Darmstadt, Germany) medium and incubated aerobically at 37 °C for 17 to 24 h at 200 rpm.

### 2.3. Identification and Taxonomic Characterization of Isolates by Sequencing

DNA from pure culture from these 111 colonies was extracted using the QIAcube RNA/DNA Extraction Instrument and the QIAamp Fast DNA Stool Mini Kit (QIAGEN, Aarhus, Denmark), following the manufacturer’s instructions. The DNA was spectrophotometrically quantified and adjusted to a final concentration of 40 ng/µL in ultrapure water (Sigma-Aldrich). The 16S rRNA gene was amplified by PCR using the oligonucleotides 1a (forward) 5′- AATACATGCAAGTCGAACGA-3′, 1b (reverse) 5′-TTAACCCAACATCTCACGAC-3′, and KOD Hot Start Master Mix (Merck KGaA, Darmstadt, Germany), following the manufacturer’s instructions. Amplicons were purified using the commercial QIAquick PCR purification kit (Qiagen Inc., Valencia, CA, USA), following the manufacturer’s instructions. Subsequent sequencing reactions were performed using the BigDye Terminator v3.1 cycle sequencing kit (premixed format; Applied Biosystems, Foster City, CA, USA). The sequencing of each amplified fragment allowed the identification of each colony at the genus and species level using the BLASTn (NCBI BLAST+ version 2.16.0) bioinformatics application against the NCBI 16S Ribosomal RNA sequences database. The strain was identified on the basis of the highest scores.

### 2.4. Lactic Acid Production

The ability of the identified vaginal lactobacillus to produce L or D-lactic acid was determined. The determinations of the two lactic acid isomers were performed using the R-Biopharm D-lactic acid/L-lactic acid kit (Boehringer Ingelheim, Darmstadt, Germany), and the UV method was used for the determination of D- and L-lactic acids in food, following the manufacturer’s instructions.

### 2.5. Activity Against Streptococcus Agalactiae

The lactobacilli of vaginal origin identified were screened to identify those that were capable of inhibiting the growth of *Streptococcus agalactiae*. The *Streptococcus agalactiae* pathogen was grown in the presence of a small amount of culture supernatant of each of the previously neutralized lactobacilli (grown in MRS medium) to determine the effect of the possible substances produced by the lactobacilli present in each of these supernatants on the growth of the pathogen. The effect of each of the supernatants tested was compared with a control culture of *S. agalactiae* to which the same amount of MRS was added, which was also neutralized. The control *S. agalactiae* growth curves and the *S. agalactiae* growth curves, with each of the neutralized supernatants obtained after the growth of each of the lactobacilli, were performed in 96-well microtiter plates in an EPOCH 2 Biotek spectrophotometer (Boston Industries, Walpole, MA, USA). Each growth was carried out in triplicate within each microtiter plate, and 3 independent experiments were carried out for each supernatant obtained. The growth curves of *S. agalactiae* were obtained by taking OD readings at 600 nm of each of the cultures that were in the wells of the microtiter plate every 2 h for 32 h in a row. The three parameters of the growth of the pathogen in culture were determined with the supernatants of the different lactobacilli, as well as the control, using the package for R, Grofit 1.1, growth velocity (µ), lag phase length (λ), and maximum cell growth (A) were evaluated.

### 2.6. CECT 7210 and CECT 30660 Cell-Free Supernatant Preparation

*B. longum* subsp. *infantis* CECT 7210 and *Lacticaseibacillus paracasei* CECT 30660 were grown for 24 h at 37 °C under anaerobic conditions on liquid MRS medium (Merck); in the case of CECT 7210, culture medium was supplemented with L-Cystein 0.05% (*w*/*v*). Cultures were centrifuged at 9000× *g* for 15 min at 4 °C, and supernatant was collected, neutralized with NaOH 10 N, and sterilized using a porous filter of 0.22 µm diameter. The cell-free supernatants were stored at −80 °C until use.

### 2.7. Animal Model

Briefly, 70 female HSD:ICR (CD-1) outbred mice (8–12 weeks old; Envigo, Sant Feliu de Codines, Barcelona, Spain) were randomly group-housed in cages with 5 mice each after an acclimatation period of one week; under a temperature-controlled environment (22 ± 2 °C), with relative humidity (55 ± 10%) and a 12 h light/dark cycle with free access to standard food and water. Female CD-1 mice were mated with males for 24 h (2 female mice per male mouse in every cage). The morning of vaginal plug detection was designated gestational day 1 (gestational length, 19–20 days), and the weight of pregnant CD-1 mice was monitored.

Intrauterine injection of LPS was given by mini-laparotomy on gestational day 15, as previously described in different studies [45,46]. Isoflurane-anesthetized mice were given analgesic buprenorphine (0.1 mg/kg), and an incision (approximately 1 cm) was made to expose the lower segments of the uterine horns. Saline solution (100 mL) or 25 µg of LPS (*Escherichia coli* 055:B5; Sigma-Aldrich, St. Louis, MO, USA) dissolved in 100 mL saline solution was injected between the two lowest gestational sacs of either the left or right uterine horn. Fascia and skin were closed with 4.0 Vicryl sutures and staples, respectively. PTB was defined as the delivery of at least 1 pup within 48 h of LPS injection.

All experimental procedures were approved by the Animal Ethics Committee of the Technological Unit of Nutrition and Health at Eurecat (Reus, Spain) and the Generalitat de Catalunya (approval number 10987). This study was conducted in compliance with the “Principles of Laboratory Care” and the European Communities Council Directive 86/609/EEC and approved 16 December 2020.

### 2.8. Effect of Intraperitoneal Injections of CECT 7210 and CECT 30660 Cell-Free Supernatant on Cytokines

Pregnant mice were distributed randomly to receive the treatments at 24 h and 30 min before the surgery. Mice were injected intraperitoneally with 200 µL saline solution or cell-free supernatant treatment (CECT 7210, CECT 30,660, or a MIX of 50% CECT 7210 and 50% CECT 30660; n = 10 per group). The sample size was chosen based on our lab’s previous successful experience and similar studies in the literature, providing reliable and reproducible results. Mice were checked at 2 h intervals for signs of PTB (vaginal bleeding, bloody bleeding, or presence of pups) for up to 12 h after surgery, and every 12 h thereafter. Animals were monitored until term for the delivery of pups, and the time of delivery was recorded. Before death, maternal blood was collected from anesthetized mice by cardiac puncture, and plasma was obtained by centrifugation at 5000× *g* for 15 min at 4 °C. The animals were sacrificed with carbon dioxide 8 h after LPS or NaCl solution intrauterine injection for the collection of amniotic fluid and placental and myometrial tissues. Amniotic fluid was pooled from all gestational sacs and centrifuged to remove any cellular debris. Placental tissue was divided from decidua and fetal membranes in ice-cold phosphate-buffered saline solution and pooled from all fetuses. Myometrium was separated from the decidua and endometrium by scraping [47]. All samples were flash-frozen in liquid nitrogen and stored at −80 °C.

### 2.9. Cytokine and Hormone Analysis

Cytokine concentrations were determined with a mouse cytokine Th17 Panel A 6-plex assay (Bio-Rad Laboratories Inc., Mississauga, ON, Canada) on a Luminex 200 cytometer and Bioplex HTF (Bio-Rad Laboratories Inc.). This assay measured concentrations of interleukin (IL)-1β, IL-6, IL-10, and IL-17A; Interferon-γ (IFN-γ); and tumor necrosis factor alpha (TNF-α). Data analysis was performed with Bio-Plex Manager software (version 5.0; Bio-Rad Laboratories Inc). Tissues were smashed and homogenized in ethylenediaminetetraacetic acid-free protease inhibitor that contained RIPA lysis buffer (1 mL per 0.5 g of tissue, Thermo Fisher Scientific Inc., Rockford, IL, USA). Homogenized samples were left on ice for 45 min before being centrifuged at 12,000× *g* for 15 min at 4 °C to finally collect the supernatant. Protein concentration was measured by Bradford assay kit (Bio-Rad Laboratories Inc.) with BSA as a standard. An amount of 250 µg of total protein was used for the measurement of cytokines in myometrium and placenta tissues. Cytokines were also analyzed in maternal plasma and amniotic fluid. Maternal plasma progesterone was measured with an Enzyme Immunoassay kit (Cayman Chemical Co., Ann Arbor, MI, USA). Maternal plasma and amniotic oxytocin were measured with an ELISA kit (Cusabio Technology LLC, Houston, TX, USA, MD, USA).

### 2.10. Statistical Analysis

Statistical analyses were performed with IBM SPSS Statistics v26 (IBM Corp., Armonk, NY, USA), using the Chi-square test, with the one-sided Fisher’s exact test used for comparison of the PTB rate; cytokines, progesterone, and oxytocin concentrations in multiple groups were analyzed with GraphPad Prism 9 software (Graph-Pad Software, La Jolla, CA, USA), using two-way analysis of variance (ANOVA) and Fisher’s exact tests for categoric variables. Data are presented as mean ± SEM. The results were expressed as means of values ± SEM, and differences between groups were set up as statistically significant when the p value was lower than 0.05.

## 3. Results

### 3.1. Characterization of Probiotic Candidate Strains

Isolation and functional characterization of *B. longum* subsp. *infantis* CECT 7210 is explained in detail in Moreno Muñoz et al., 2011 [48]. Following the methodology described in Section 2, 111 colonies isolated from vaginal exudate samples collected with sterile swabs of healthy women presented morphology of elongated bacilli with Gram-positive bacteria; a total of 55 bacteria were identified as members of the *Lactobacillus* genus, and one of these lactobacilli strains, internally designated as *L. paracasei* ORD 0998, was deposited in the Spanish Type Culture Collection (CECT) with the reference *L. paracasei* CECT 30660.

Table 1 shows the production of the two lactic acid isomers, D-lactic acid and L-lactic acid, for some of the lactobacilli isolated, total lactic acid produced, and the percentage of L-lactic acid vs. total lactic acid produced. *Lc. paracasei* ORD0 998 (CECT 30660) can produce 72.63 ± 8.27 mM of total lactic acid, being 94 ± 5.06% L-lactic acid (68.30 ± 6.61 mM of L-lactic acid and 4.34 ± 3.76 mM of D-lactic acid). These results show that *L. paracasei* ORD 0998 (CECT 30660) is a good lactic acid-producing bacterium, especially of L-lactic acid.

In addition, Table 2 shows the growth of *S. agalactiae* in the presence of lactobacilli supernatant. Absorbance at 660 nm (A_660_) vs. time data of the culture of *S. agalactiae* with lactobacilli supernatant have allowed us to obtain, using the Grofit R package (version 1.1.1-1), the growth speed (µ) and the maximum cell growth (A_660 max_) of this pathogen standardized with respect to the control growth curve of the pathogen. The results obtained allowed us to establish the inhibition capacity of each of the lactobacilli supernatants used; the greater the inhibition, the lower the normalization of the µ and A_660 max_ obtained for the culture of *S. agalactiae*.

### 3.2. The Combination of CECT 7210 and CECT 30660 Cell-Free Supernatant Reduced LPS-Induced PTB in Mice

**Figure 1:**
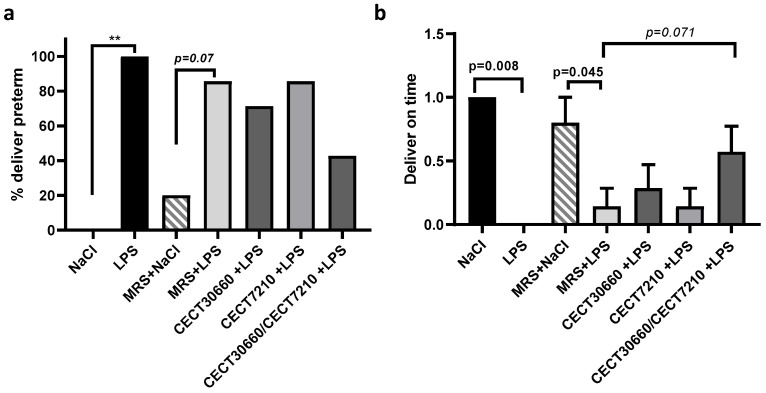
Combined treatment of two cell-free supernatants reduced the percentage of preterm deliveries (**a**) and increased the percentage of on-time deliveries (**b**). (**a**) Group NaCl showed 0% preterm deliveries, whereas LPS injection caused 100% PTB. The MRS + LPS, ORD + LPS, and CECT + LPS groups slightly reduced the % of preterm deliveries. However, the combination of both cell-free supernatants reduced the effect of LPS injection on preterm deliveries. (**b**) Representation of on-time term deliveries (value = 1) or premature deliveries (value = 0) of the same groups of animals. n = 10/group. ** *p* < 0.01.

The effect of the different treatments on PTB was analyzed through the percentages of preterm deliveries and on-time deliveries in the animal model (Figure 1). Preterm labor was not induced in animals treated intrauterine and intraperitoneally with saline. On the contrary, animals treated intrauterine with LPS and intraperitoneally with saline produced 100% of the premature deliveries (Figure 1a). Similar results were obtained with the animals treated with the bacterial growth medium MRS by IP and treated intrauterine (NaCl (20%) vs. LPS (85.6%)). Animals treated intrauterine with LPS and individually by IP with CECT 30660 or CECT 7210 presented a little effect on preterm deliveries (71.4% and 85.6%, respectively). In contrast, the combination of both cell-free supernatant administered intraperitoneally reduced preterm deliveries by up to 42.8% (Figure 1a). The percentage of preterm deliveries was contrasted with the percentage of on-time deliveries, which were negatively correlated (Figure 1b). To gain a deeper understanding of the potential role of the cell-free supernatant combination in the reduction in preterm labor, analysis of cytokine inflammation levels and other hormones was performed in a new group of animals treated intrauterine with saline or LPS and injected intraperitoneally with saline or the cell-free supernatant MIX.

### 3.3. The Combination of CECT 7210 and CECT 30660 Cell-Free Supernatants Attenuated LPS-Induced Cytokine Levels

**Figure 2:**
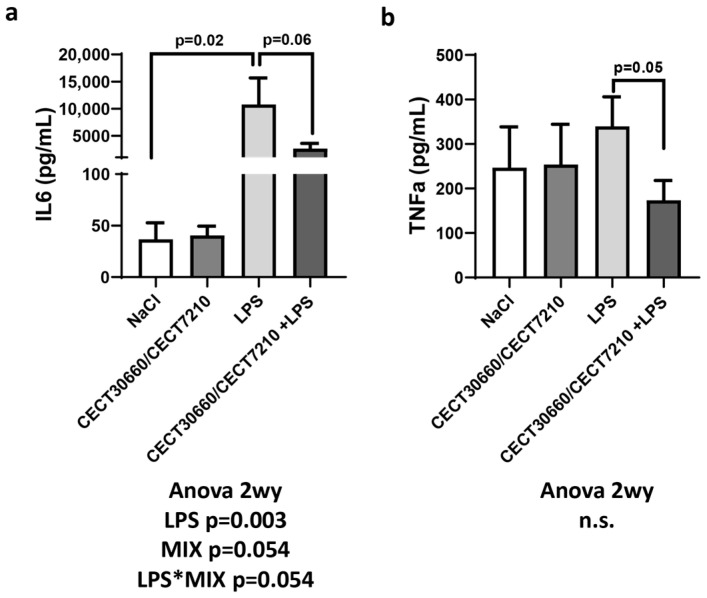
Plasma concentrations of proinflammatory cytokines interleukin 6 (IL-6) (**a**) and tumor necrosis factor alpha (TNF-α) (**b**) after treatments. n.s.: non-significant. “*” in “LPS*MIX” means interaction. n = 10/group.

The LPS intrauterine injection plus saline IP injection group (LPS group) resulted in a significant increase in the plasma proinflammatory cytokines IL-6 (Figure 2) and IL-10 (Figure S1). In addition, animals treated with intrauterine LPS injection that were previously treated with the MIX of cell-free supernatants (MIX + LPS group) showed an important decrease in maternal plasma levels of IL-6 and TNF-α (Figure 2). No differences were observed in the IFN-γ and IL-17 α levels (Figure S1). In contrast, no significant changes were found in the analysis of cytokines between groups in the amniotic fluid (Figure S2).

**Figure 3:**
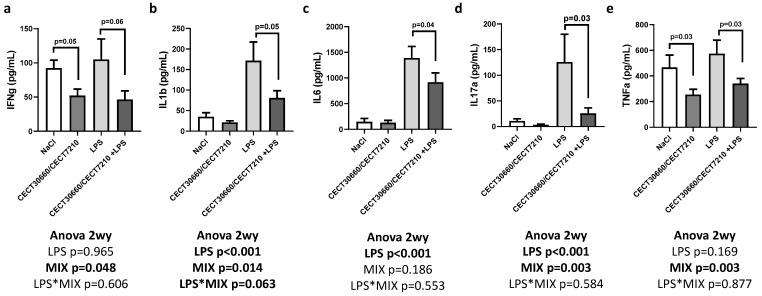
Myometrium concentrations of proinflammatory cytokines after treatments: interferon gamma (IFN-g) (**a**); interleukin 1 beta (IL1-b) (**b**); interleukin 6 (IL-6) (**c**); interleukin 17 α (IL-17a) (**d**); and tumor necrosis factor alpha (TNF-α) (**e**). “*” in “LPS*MIX” means interaction. n = 10/group.

On the other hand, the analysis of cytokine levels in the myometrium showed some significant changes in their concentrations (Figure 3). The MIX group showed an important decrease in IFN-γ levels. In addition, treatment with the cell-free supernatant MIX caused a significant decrease in the myometrium cytokine levels of IL-1β, IL-6, TNF-α, and IL-17α compared to the LPS group (Figure 3). Cytokine levels were also analyzed in the placenta, showing an increased level of IL-1β and IL-6 after intrauterine LPS treatment (Figure S3). However, treatment with MIX injection did not affect IL-1β and IL-6 placental levels. No significant changes were observed in placental TNF-α levels after different treatments.

### 3.4. Effect of the Combination of CECT 7210 and CECT 30660 Cell-Free Supernatants on Maternal Plasma Progesterone Concentration and Oxytocin Levels

Maternal plasma progesterone level was analyzed after different treatments (Figure S4a). LPS treatment significantly reduced maternal progesterone concentration. The saline group that received the CECT 7210 and CECT 30660 supernatant MIX maintained high concentrations of plasma progesterone in comparison with the saline group without IP treatment. To confirm that these concentrations are identical between groups or that the mixed supernatant IP injection may cause any effect, a powerful analysis test should be required to demonstrate differences. Maternal plasma oxytocin and amniotic oxytocin concentration were analyzed after different treatments. The MIX + LPS group presented a significant decrease compared to the MIX group in maternal plasma oxytocin levels (Figure S4b,c). However, no other significant changes were observed between other groups in maternal plasma or amniotic oxytocin levels.

## 4. Discussion

Probiotics are defined by their demonstrated health-promoting effects in the host, which may include, among other mechanisms, the ability to inhibit the growth of pathogenic bacteria. In this study, we observed that the combination of two cell-free supernatants from CECT 7210 and CECT 30660 reduced LPS-induced PTB and ameliorated systemic inflammation in pregnant mice. The first strain, in combination with other pre- and probiotics, has a positive effect against pathogens by reducing harmful bacterial species in the gastrointestinal mucosa and fecal excretion due to a successful colonization in the gut [42,49]. In addition, CECT 7210 has been shown to be capable of inhibiting the growth of a large number of Gram-negative pathogens, preventing the adhesion of some pathogens relevant to bacterial vaginosis [50]. However, these strains meet some but not yet all criteria for probiotic designation. CECT 30660 is selected in this work, given its good production of L-lactic acid and its ability to inhibit the growth of other Gram-positive bacteria relevant to PTB. However, the most interesting characteristic of CECT 7210 is the anti-inflammatory properties, such as promotion of maturation of the immune response, reduction in intestinal permeability, and production of short-chain fatty acids (SCFAs) [39], which could help to prevent PTB caused by inflammatory processes [51]. CECT 7210 ameliorates inflammatory processes caused by tight junction protein destabilization through the production of indole-3-lactic acid (ILA), which attenuates LPS-induced activation of NF-kB in macrophages [52]. After birth, CECT 7210 has an important role during breastfeeding due to its ability to metabolize human milk oligosaccharides and produce SCFAs, giving beneficial metabolic effects [39].

PTB may be a consequence of physiological inflammation coordinated by multiple uterine tissues (fetal and maternal) [53]. These inflammatory stimuli consist of activation of innate immunity via toll-like receptors (TLRs), which leads to cytokine production and initiates an inflammatory response related to leukocyte activation and transmigration. This effect is mediated by some cytokines, such as interleukin IL-6, (IL)-1, and TNFα, among others [54]. LPS stimulated increased levels of cytokines, producing a systemic and intrauterine inflammation, which was expected, bearing in mind the results of previous studies using the same PTB mouse model [20,47]. We administered cell-free supernatants individually and combined before LPS injection because it is likely that the protective benefit in humans would be in the prevention of PTB. We suggest that the active moiety in CECT 7210 and CECT 30660 cell-free supernatants, when given intraperitoneally, activate signaling molecules that trigger immune modulatory responses and effects that have been observed. The mouse PTB model is a valuable tool for studying mechanisms and potential treatments, such as cell-free probiotic supernatants. However, differences in gestation, placental structure, and physiology between mice and humans limit direct translation. Thus, while findings provide important mechanistic insights, further validation in human studies is needed to confirm clinical relevance.

Some cytokines were profiled in the maternal plasma, myometrium, amniotic fluid, and placenta after LPS injection and in combination with the cell-free supernatant MIX. LPS induced the more notorious changes in myometrium and maternal plasma, where proinflammatory cytokine concentrations were altered, increasing their levels significantly. This affection in the myometrial tissue plays a key role in the LPS-induced preterm delivery, suggesting immune alterations, such as immune cell infiltration in the myometrium, which may contribute to the process of PTB [47]. The observed absence of significant cytokine modulation in the placenta and amniotic fluid, despite clear anti-inflammatory effects in the myometrium, suggests distinct tissue-specific immune environments and differential bioavailability of postbiotic metabolites, highlighting the complexity of local immune regulation during pregnancy [47]. Although these results are consistent with mouse model studies, they cannot be extrapolated directly to human clinical studies. However, many of the cytokines we have reported are implicated in the pathogenesis of human PTB [53,55].

The combined treatment of cell-free supernatants gave evidence that promotes an anti-inflammatory effect against LPS-induced inflammation. This anti-inflammatory activity is consistent with some bifidobacterial studies. Some studies in the gastrointestinal tract described a down-modulation of proinflammatory cytokines, blocking LPS-induced NF-kB activation [56,57]. Moreover, a study with maternal immune activation where pregnant mice were fed orally until birth with probiotic *Bifidobacterium infantis* combined with *B. bifidum* reported a significant decrease in maternal circulating cytokine levels [58]. Our mixed treatment with cell-free supernatants especially decreased systemic LPS-induced cytokine concentrations. TNF-α and IL-6 concentrations were diminished significantly in myometrium and maternal plasma, but this effect was not observed in the placenta and amniotic fluid. In consonance with our results, in a study with pregnant mice with knockout for IL-6 [59], PTB was delayed in comparison with control mice, and TNF-α and IL-1β double-knockout mice are resistant to bacterially induced PTB [60]. These findings suggest that both TNF-α and IL-6 have a huge importance in PTB when it is induced by LPS.

IL-1β is related to human PTB [54]. IL-1β was found to be increased in an LPS-induced PTB murine model, but PTB was ameliorated by decreasing IL-1β circulating levels through a small non-competitive IL-1R-biased ligand [29]. In our study, IL-1β levels were increased in myometrium and placenta due to LPS injection, but were meaningfully reduced in myometrium through the injection of our combination of cell-free supernatants. In the case of IL-10, it is an interleukin that promotes anti-inflammatory responses, inhibiting inflammatory cytokine synthesis like IL-6 and TNF-α [30]. Thus, in a study of an IL-10^−/−^ mice model injected with LPS, proinflammatory cytokines were found to increase, reducing gestation time, fetal growth restriction, and fetal loss [30]. Increased levels of IL-10 were not significantly associated with PTB [24]. This is consistent with our study because IL-10 does not show any significant change between groups. Another important cytokine that has a key role in inflammatory processes is IL-17 α, which is implicated in the pathogenesis of PTB [61]. We observed a significant increase in myometrial IL-17 α levels due to LPS injection, which is a consistent outcome based on the results obtained in a study of an LPS-induced PTB mouse model [20]. However, the LPS effect on myometrial IL-17 α levels was reduced through combined administration of CECT 7210 and CECT 30660 supernatants, which could help to positively normalize gestational term and reduce PTB. Finally, a study demonstrated that an increase in IFN-γ levels causes contractility of human myometrial cells, initiating preterm uterine contractions [25]. This outcome is related to our results in the myometrium, which presented significantly elevated levels of IFN-γ due to LPS treatment. This affection was ameliorated through the IP injection of the MIX of cell-free supernatants, which showed an important decrease in IFN-γ levels related to the reduction in PTB. Both amniotic and plasma IFN-γ levels showed a tendency to be reduced through combined CECT 7210 and CECT 30660 injection.

Progesterone is considered a key factor in pregnancy maintenance in humans via promoting uterine quiescence because its inhibitory action could trigger preterm labor [31,62]. Progesterone withdrawal is important to initiate murine labor, which is concordant with clinical studies. However, the LPS-induced PTB mouse model can activate inflammation and may be sufficient to annul the repressive effects of high circulating levels of progesterone, which decline if progesterone is essential to undergo PTB [63]. Plasma progesterone concentrations showed decreased levels through LPS injection. This significant LPS-induced fall in circulatory progesterone levels is consistent with a previous study with PTB mice models [27,64]. These results propose inflammation as a more important key factor compared to the progesterone withdrawal effect.

Oxytocin is a hormone that induces contraction and is related to parturition. Elevated levels of progesterone inhibit the responsiveness of myometrial cells to oxytocin, maintaining cervical integrity. Progesterone withdrawal leads to up-regulation of the oxytocin receptor, which increases the responsiveness of the myometrial cells to oxytocin [34]. In our study, oxytocin did not present any significant change in amniotic fluid. The lack of significant changes in oxytocin levels could be linked to the absence of meaningful changes in progesterone levels. In order to confirm this theory, an analysis of oxytocin levels and the expression of the oxytocin receptor in myometrium could be interesting due to the relation of LPS-induced PTB and the elevated levels of this hormone and the up-regulation of its receptor in this tissue observed in other studies [65]. Future steps should include the complete characterization of the bioactive components present in the cell-free supernatants through targeted metabolomic analyses. Moreover, it is also critical to decipher the molecular mechanisms of this improvement, specifically to evaluate the role of TLR signaling and NF-κB regulation. Additionally, while the in vivo results are promising, it is necessary to validate the safety and efficacy of the cell-free supernatant administered orally through controlled in vivo animal models and human clinical trials. Such studies are essential to confirm the biological effects observed in this study and evaluate potential therapeutic applications in humans.

## 5. Conclusions

In summary, this study provides robust preclinical evidence that cell-free supernatants derived from B. longum *Bifidobacterium longum* subsp. *infantis* CECT 7210 and *Lacticaseibacillus paracasei* CECT 30660 can attenuate LPS-induced PTB in mice by modulating proinflammatory cytokines in maternal plasma and the myometrium. This protective effect appears to be independent of changes in progesterone and oxytocin levels. The findings highlight a novel anti-inflammatory strategy with potential application in obstetric care and support the further development of cell-free supernatant as a therapeutic agent for reducing the risk of preterm labor. Additional mechanistic and clinical studies are warranted to translate these promising findings into human health benefits.

## Figures and Tables

**Table 1:** Production of lactic acid by isolated lactobacilli.

Species	L-Lactic Acid (mM)	D-Lactic Acid (mM)	Total Lactic Acid (mM)	% L-Lactic Acid
*L. rhamnosus GG*	63.60 ± 4.52 ^a^	4.02 ± 1.01 ^a^	67.62 ± 3.51 ^a^	94 ± 1.81% ^a^
*L. gasseri* ORD 0774	31.95 ± 1.04 ^b^	44.09 ± 7.49 ^b,c^	76.05 ± 6.55 ^a,b^	42 ± 4.97% ^b^
*L. gasseri* ORD 0775	29.59 ± 2.95 ^b^	45.58 ± 4.58 ^b,c^	75.16 ± 7.32 ^a,b^	39 ± 1.08% ^b^
*L. crispatus* ORD 0795	16.43 ± 0.63 ^b^	57.05 ± 3.23 ^b,c^	73.48 ± 3.78 ^a,b^	22 ± 0.54% ^b^
*L. crispatus* ORD 0798	16.40 ± 2.90 ^c^	70.72 ± 4.44 ^b,c^	87.11 ± 7.08 ^b,c^	19 ± 1.96 % ^b^
*L. rhamnosus* ORD 0856	61.73 ± 1.36 ^a,d^	0.76 ± 0.82 ^a,d^	62.49 ± 1.95 ^a,d^	99 ± 1.29% ^a,c^
*L. crispatus* ORD 0862	13.13 ± 2.42 ^c^	17.77 ± 7.30 ^c,e^	30.90 ± 8.71 ^d,e^	44 ± 9.59% ^b^
*Lc. paracasei* ORD 0873	65.18 ± 14.04 ^a,e^	4.95 ± 0.77 ^a,e^	70.13 ± 14.66 ^a,b,d^	93 ± 0.83 % ^a,c^
*Lc. paracasei* ORD 0960	40.12 ± 4.29 ^f^	2.45 ± 2.14 ^a,d^	42.58 ± 6.42 ^d,e^	95 ± 4.61% ^a,c^
*Lc. paracasei* ORD 0966	44.39 ± 5.44 ^b,f^	0.00 ± 0.00 ^d^	44.39 ± 5.44 ^d^	100 ± 0.00% ^c,d^
*Lc. paracasei* ORD 0968	49.76 ± 8.14 ^d,f^	4.09 ± 4.36 ^a,d^	53.85 ± 12.14 ^a,d^	93 ± 6.78% ^a,c^
*Lc. paracasei* ORD 0989	70.70 ± 9.96 ^a,d,e^	2.41 ± 3.17 ^a,d^	73.11 ± 8.20 ^a,b,c^	97 ± 4.69% ^a,c^
*Lc. paracasei* ORD 0993	74.77 ± 3.65 ^e^	4.55 ± 1.49 ^a,d^	79.32 ± 3.09 ^b,c^	94 ± 1.98% ^a,c^
*Lc. paracasei* ORD 0998 (CECT 30660)	68.30 ± 6.61 ^a,d,e^	4.34 ± 3.76 ^a,d^	72.63 ± 8.27 ^a,b,d^	94 ± 5.06% ^a,c,d^

Lactic acid production data after 18 h of incubation. Within the same column, the letters represent sets where there are no significant differences between the data according to the Conover–Iman test. Correction *p* value = fdr.

**Table 2:** Growth of *S. agalactiae* in the presence of lactobacilli supernatant.

Strain	Growth Velocity (μ Normalized)	Maximum Cell Growth (A_660 Max._ Normalized)
*L. gasseri* ORD 0793	0.767 ± 0.117 *	0.773 ± 0.070 *
*L. gasseri* ORD 0847	0.726 ± 0.144 **	0.723 ± 0.118 **
*L. gasseri* ORD 0854	0.820 ± 0.144	0.766 ± 0.053 **
*L. crispatus* ORD 0858	0.789 ± 0.137	0.747 ± 0.085 **
*L. crispatus* ORD 0868	0.800 ± 0.106 *	0.834 ± 0.094 *
*L. crispatus* ORD 0973	0.727 ± 0.086 **	0.734 ± 0.079 **
*Lc. paracasei* ORD 0986	0.752 ± 0.084 *	0.783 ± 0.045 *
*Lc. paracasei* ORD 0993	0.704 ± 0.091 **	0.730 ± 0.079 **
*Lc. paracasei* ORD 0998 (CECT 30660)	0.685 ± 0.104 **	0.712 ± 0.075 **

Growth velocity (µ) and maximum cell growth (A_660 Max._) standardized regarding the control. *: *p* < 0.05. **: *p*′ < 0.05, where *p*′ = *p*/No. Ho Dunn Test (Bonferroni’s Correction).

## Data Availability

The data that support the findings of this study are available from the corresponding authors upon reasonable request.

## Supplementary Materials

The following supporting information can be downloaded at https://www.mdpi.com/article/10.3390/nu17213429/s1: Figure S1: Plasma concentrations of proinflammatory cytokines: (a) interleukin 10 (IL10); (b) interferon gamma (IFNg) and interleukin 17a (IL17a) (c) after treatments. n.s.: non-significant. n = 10/group. Figure S2: Amniotic fluid concentrations of proinflammatory cytokines after treatments: interferon gamma (IFNg) (a); interleukin 6 (IL6) (b); tumor necrosis factor alpha (TNFa) (c); and interleukin 17a (IL17a) (d). n.s.: non-significant. n = 10/group. Figure S3: Placenta concentrations of proinflammatory cytokines after treatments: interleukin 1b (IL1b) (a); interleukin 6 (IL6) (b); and tumor necrosis factor alpha (TNFa) (c). n.s.: non-significant. n = 10/group. Figure S4: Maternal plasma progesterone (a); oxytocin in plasma (b); and oxytocin in amniotic fluid (c) concentrations after the different treatments. n.s.: non-significant. n = 10/group.
