# Supplementary material for: A Combination of Lacticaseibacillus paracasei CECT 30660 and Bifidobacterium longum subsp. infantis CECT 7210 Cell-Free Supernatants Reduces LPS-Induced Preterm Birth and Systemic Inflammation in Pregnant Mice
Source: Nutrients. 2025 Oct 31;17(21):3429. doi: 10.3390/nu17213429 (PMC12609628; doi:10.3390/nu17213429)
Supplement: Supplementary file 1 [file nutrients-17-03429-s001.zip › nutrients-3912155-supplementary.pdf]

FigS1

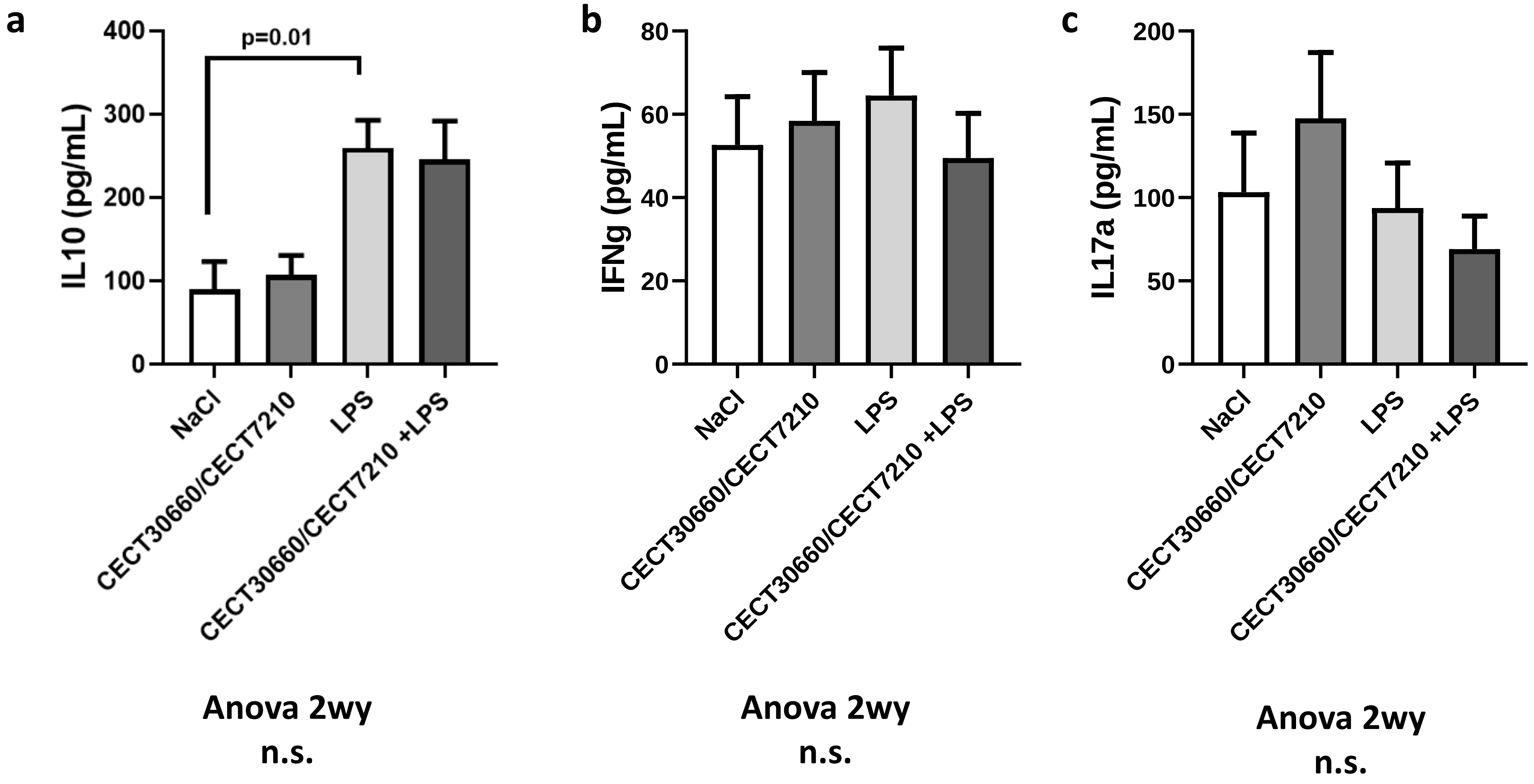

Figure S1. Plasma concentrations of proinflammatory cytokines interleukin 10 (IL10) (**a**) interferon gamma (IFNg) (**b**) and interleukin 17a (IL17a) after treatments (**c**). n.s.; non-significant.

FigS2

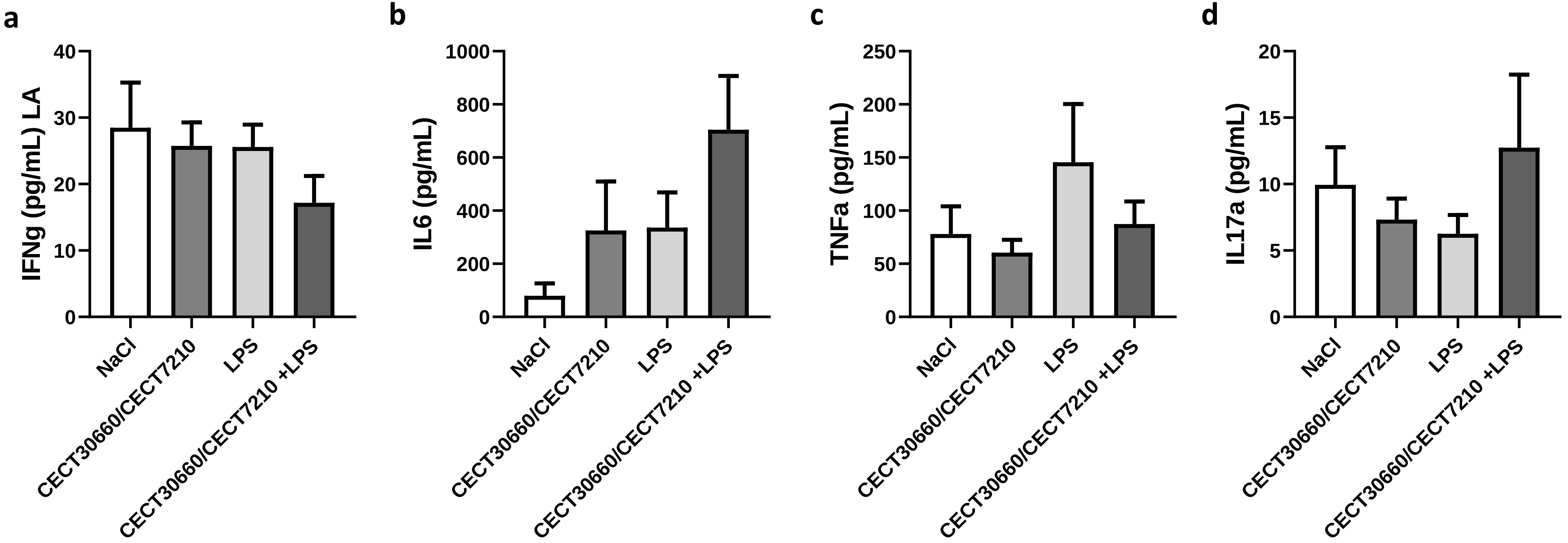

Anova 2wy
n.s.

Anova 2wy
LPS p=0.080 MIX p=0.090
LPS × MIX p=0.731

Anova 2wy
n.s.

Anova 2wy
n.s.

Figure S2. Amniotic fluid concentrations of proinflammatory cytokines after treatments: interferon gamma (IFNg) (**a**); interleukin 6 (IL6) (**b**); tumor necrosis factor alpha (TNFa) (**c**); and interleukin 17a (IL17a) (**d**). n.s.; non-significant.

FigS3

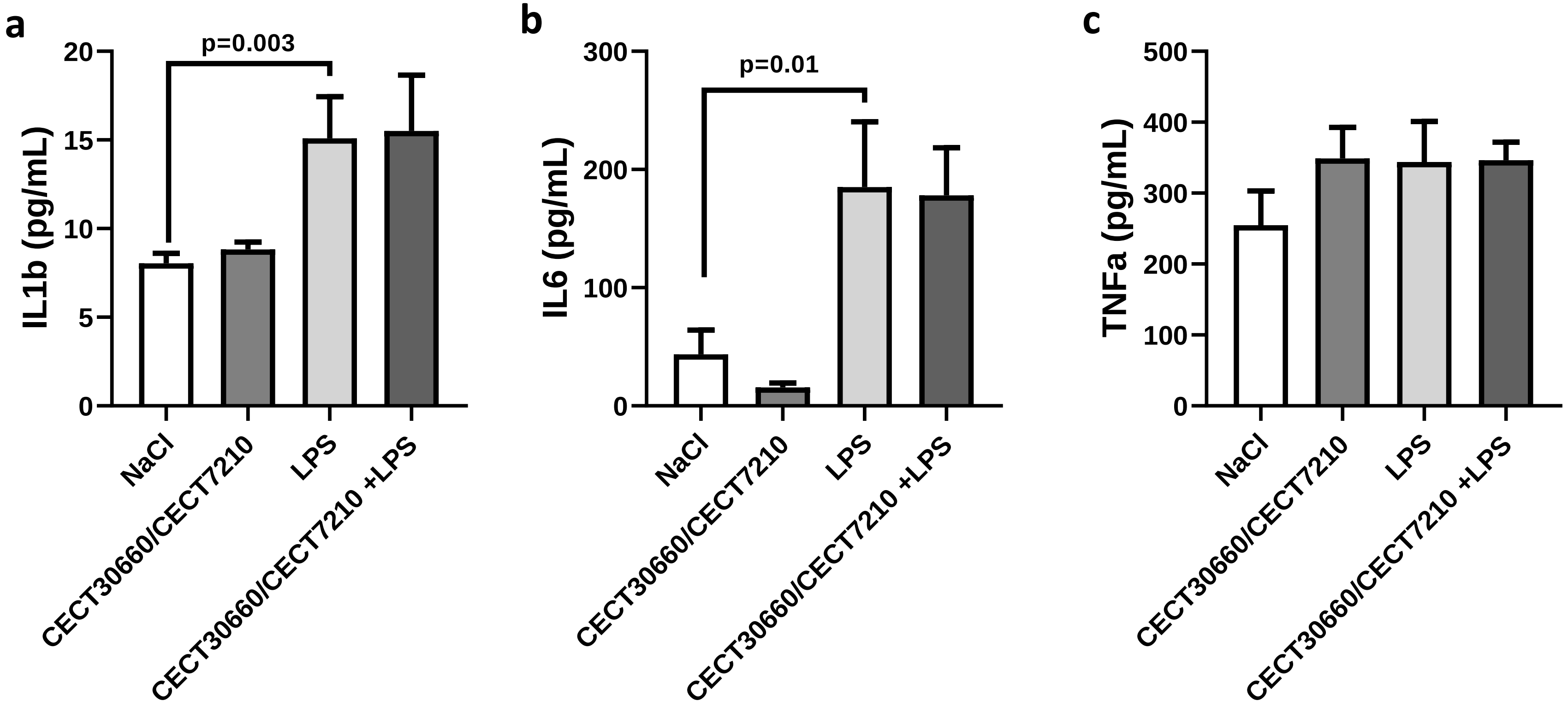

Anova 2wy
LPS p<0.001 MIX
p=0.854
LPS × MIX p=0.529

Anova 2wy
LPS p<0.001 MIX
p=0.741
LPS × MIX p=0.725

Anova 2wy
n.s.

Figure S3. Placenta concentrations of proinflammatory cytokines after treatments: interleukin 1b (IL1b) (**a**); interleukin 6 (IL6) (**b**); and tumor necrosis factor alpha (TNFa) (**c**). n.s.; non-significant.

FigS4

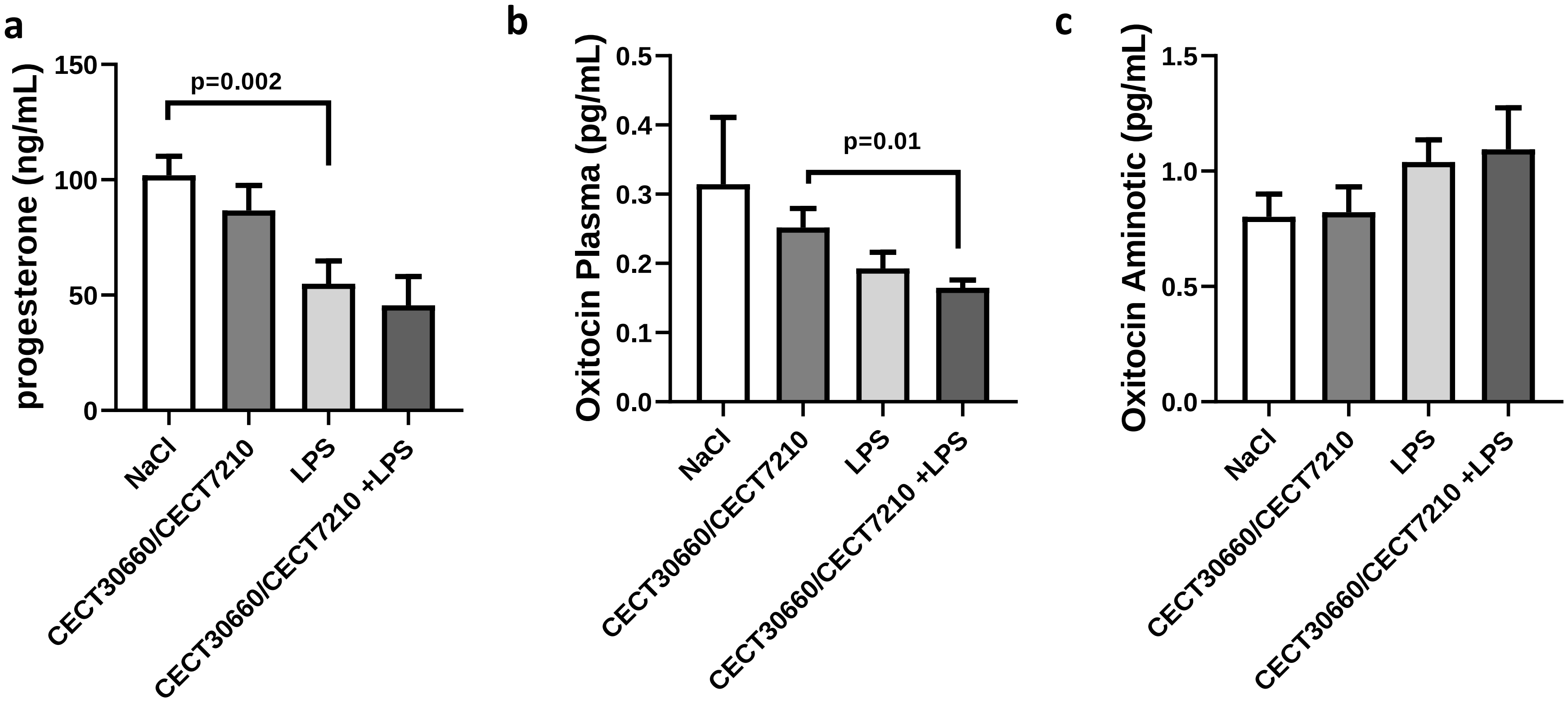

Anova 2wy
LPS p<0.001 MIX
p=0.268
LPS × MIX p=0.793

Anova 2wy
n.s.

Anova 2wy
n.s.

Figure S4. Maternal plasma progesterone (**a**); oxytocin in plasma (**b**) and oxytocin in aminiotic fluid (**c**) concentrations after the different treatments. n.s.; non-significant.
